# Analysis of the changes in health-related quality of life and employment status after surgery in patients with lung cancer: a single-center longitudinal study

**DOI:** 10.1007/s11748-025-02144-z

**Published:** 2025-04-20

**Authors:** Yuka Kadomatsu, Toru Oga, Atsuhiko Ota, Hiroshi Yatsuya, Yuta Kawasumi, Harushi Ueno, Taketo Kato, Shota Nakamura, Tetsuya Mizuno, Toyofumi Fengshi Chen-Yoshikawa

**Affiliations:** 1 Department of Thoracic Surgery, Nagoya University Graduate School of Medicine, 65 Tsurumai-cho, Showa-ku, Nagoya, 466-8550 Japan; 2 Department of Respiratory Medicine, Kawasaki Medical School, Kurashiki, Okayama Japan; 3 Department of Public Health, School of Medicine, Fujita Health University, Toyoake, Aichi Japan; 4 Department of Public Health and Health Systems, Graduate School of Medicine, Nagoya University, Nagoya, Japan

**Keywords:** Return to work, Lung neoplasms, Health-related quality of life, Retirement, EORTC

## Abstract

**Objective:**

This study analyzed the changes in health-related quality of life (HRQOL) and employment status of patients undergoing lung cancer surgery in Japan.

**Methods:**

This was a single-center, prospective study on patients who underwent lung anatomical resection. The eligible patients completed self-reported HRQOL and employment surveys at baseline and 6 and 12 months postoperatively. HRQOL was assessed using questionnaires including the European Organization for Research and Treatment of Cancer Core Quality of Life questionnaire (EORTC QLQ-C30) and EORTC QLQ and Lung Cancer module and additional social engagement and work-related stress evaluation tools.

**Results:**

In total, 93 patients completed the baseline survey, and 80 provided survey data at 6 months postoperatively. The HRQOL scores of several factors significantly declined immediately after the surgery and then gradually improved. The EORTC global health score, which represents overall health status, returned to baseline levels at 12 months postoperatively. However, symptoms such as fatigue, dyspnea, and coughing did not return to baseline levels at 12 months postoperatively. Approximately 68% of the patients who were employed preoperatively continued to work at 12 months postoperatively.

**Conclusions:**

Lung cancer surgery significantly affected the HRQOL and employment status of the patients within the first 6 months after surgery. For patients who decide to return to work before full recovery of QOL, we consider the need for enhanced support to assist them as they can reintegrate into work and activities of daily living.

## Introduction

Lung cancer has a higher mortality rate than other types of cancer. Therefore, its treatment aims to significantly prolong the survival of patients [1]. Complete surgical resection is the only potentially curative treatment option; therefore, patients have tended to undergo lung surgery at the highest acceptable intensity [2]. On the other hand, since the lung is considered a non-regenerative organ, postoperative loss of lung function also affects the patients’ daily activities [3]. Recently, with advancements in perioperative management and new treatments like immune checkpoint inhibitors, outcomes for patients with lung cancer have improved [1, 4]. As survival rates have increased, attention has shifted toward their life courses and work following lung cancer treatment. Health-related quality of life (HRQOL) has been incorporated into the World Health Organization recommendations as a key component of patient assessment prior to lung cancer treatment. Thereafter, as observed globally, increased attention has been paid to treatment-related impact on HRQOL [5]. Although several meta-analyses of the effects of lung cancer surgery on HRQOL have been conducted worldwide, such reports remain limited in Japan due to the delay in the introduction of measuring HRQOL based on linguistic differences. [6, 7]. Consequently, the changes in patient reported outcomes such as HRQOL after lung cancer treatment are not fully revealed in Japan.

Cancer treatment negatively impacts employment and social participation to varying degrees [8, 9]. Retirement due to cancer treatment is not only an issue for individuals but also a loss for society due to shortage in the labor force. Employment support for patients with cancer is an important feature in the Basic Plan to Promote Cancer Control Programs [10]. The Japanese survey of return-to-work (RTW) rate of cancer survivors from large-scale companies showed that the RTW rate was relatively low in patients with lung cancer, and the 1-year RTW rate was approximately 75.3% [11], although it differed based on cancer type [11, 12]. A company-based study offers the advantage of examining detailed employment-related information; however, it lacks detailed medical information and data on many patients working at small- and medium-sized enterprises. The working-age population, typically those aged 15–64, has been declining, while the number of workers aged 65 and older is increasing. In general, the incidence of lung cancer rises among individuals aged 60 years and older [13]. Therefore, we analyzed baseline profiles and post-operative changes in HRQOL and work- and social-related activities in patients who underwent lung cancer surgery in Japan.

## Methods

### Study population

**Fig. 1:**
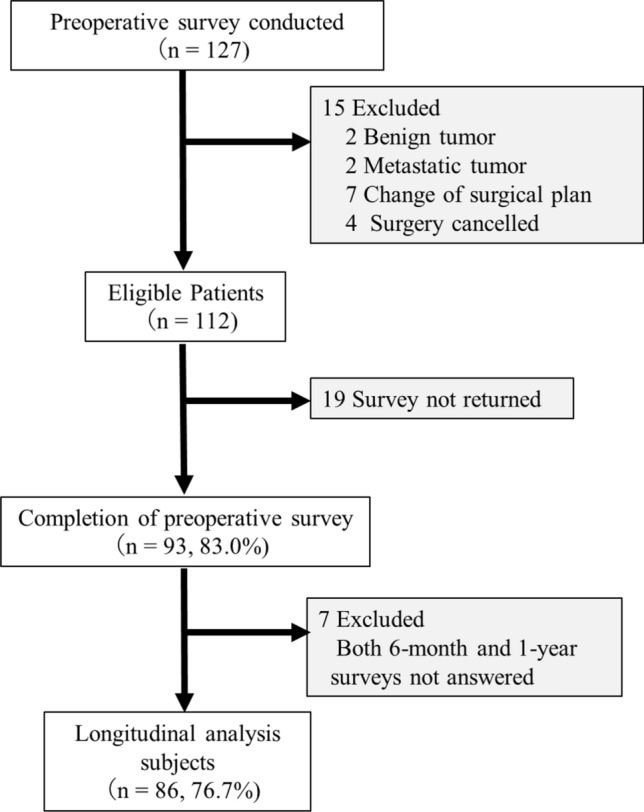
Patient enrollment and follow-up. A total of 127 patients completed the preoperative survey, of whom 15 were excluded for not meeting inclusion criteria. Of the remaining 112 eligible patients, 93 (83.0%) completed the preoperative survey, and 86 (76.7%) provided data for longitudinal analysis after excluding those who did not complete both the 6-month and 1-year follow-up surveys

We prospectively recruited patients who underwent lung anatomical resections for primary lung cancer at Nagoya University Hospital between January 2022 and September 2022. Figure 1 shows a flow chart summarizing the number of patients included in this study. The major exclusion criteria were patients with non-lung cancer, those who underwent non-anatomic resection, those scheduled for simultaneous resection of adjacent organs, those who underwent non-elective surgery, and those who lacked adequate Japanese language skills.

### Patient-reported outcomes and survey methods

Patients who were invited to join this study completed a survey on basic social economic status and several self-reported assessments. They participated in surveys before surgery, at the initial postoperative visit, and at 6 and 12 months after the surgery. The baseline survey was generally performed 1 month before the surgery, and the initial visit after the surgery was scheduled approximately 1.5 months after the surgery. The 6- and 12-month postoperative surveys were conducted via mail.

The basic information included questions on final education level, marriage, household composition and income, employment status, type of employment, and the attributes and size of the occupation if employed. The following items were assessed with corresponding questionnaires. Evaluation of stress in the workplace—Demand–Control–Support Questionnaire (DCSQ-17) [14]; degree of social participation—JST–Index of Competence (JST–IC) [15]; patient perspective on hospital care—Hospital Consumer Assessment of Healthcare Providers and Systems (HCAHPS) [16]; HRQOL—the European Organization for Research and Treatment of Cancer Core Quality of Life Questionnaire (EORTC QLQ-C30 version 3.0) and its lung cancer-specific module, the Lung Cancer Module (LC-13) [17, 18], and MOS Short-Form 36-Item Health. Survey (SF-36v2) [19, 20]; depression scale—Center for Epidemiologic Studies Depression Scale (CES-D) [21]; sleep quality-Pittsburgh Sleep Quality Index (PSQI) [22]; and social isolation—Lubben Social Network Scale (LSNS) [23]. Table 1 shows the details of the questionnaire assessing each item and the timing of administration. Comorbidities were assessed by the Charlson Comorbidity Index (CCI) [24].

**Table 1:** Details of the conducted patient-reported questionnaires

	Before surgery	Time of discharge	Initial outpatient visit	6 months after surgery	12 months after surgery
Basic information	〇			〇	〇
DCSQ-17^13)^	〇			〇	〇
Social participation (Part of JST-IC)^14)^	〇			〇	〇
HCAHPS^15)^		〇			〇
EORTC^16,17)^	〇		〇	〇	〇
SF-36^18,19)^	〇		〇	〇	〇
CES-D^20)^	〇		〇	〇	〇
PSQI^21)^	〇		〇	〇	〇
LSNS^22)^	〇		〇	〇	〇

*CES-D* Center for Epidemiologic Studies Depression Scale, *DCSQ-17* Demand-Control-Support Questionnaire, *EORTC* European Organization for Research and Treatment of Cancer, *PSQI* Pittsburgh Sleep Quality Index, *HCAHPS* Hospital Consumer Assessment of Healthcare Providers and Systems, *JST-IC* Japan Science and Technology Agency Index of Competence, *LSNS* Lubben Social Network Scale, *SF-36* Short Form Health Survey

### Details of the research tools

In the present study, we focused on the DCSQ, JST–IC, EORTC QLQ–C30, and LC13. DCSQ consists of 17 items designed to assess work-related stress across four domains: psychological demand, decision latitude–skill discretion (DL–SD), decision latitude–decision authority (DL–DA), and social support [14]. The JST–IC scale was developed to assess higher-level functional capacity in older adults, and it includes 16 items across four domains. In this study, only the Social Engagement domain was assessed, with participants rating their independence on a dichotomous scale (0 = no, 1 = yes) [15]. Each item is rated on a four-point scale, and the scores in each respective domain were summed. The EORTC QLQ–C30 and QLQ–LC13 are standardized questionnaires developed by a European organization to assess HRQOL in patients with cancer. The QLQ–C30 evaluates the general aspects of HRQOL including physical, emotional, and social functioning. Meanwhile, the LC13 module specifically addresses lung cancer-related symptoms. The scores of each domain and the treatment of missing values were calculated based on the formal scoring manual. Higher scores on the GHS and QLQ–C30 functional scales indicate better condition.

### Ethics approval

The study protocol was approved by the Institutional Review Board of Nagoya University School of Medicine (2017–0034, May 11, 2017). A written informed consent was obtained from all the patients.

### Statistical analysis

Results were presented mean ± standard deviation (SD). Categorical variables were presented as counts and percentages. Continuous variables were compared using the Mann–Whitney U or the Student’s *t* test after assessing the normality of data. Categorical variables were compared using Pearson’s chi-square or the Fisher’s exact test. RTW was defined as returning to a job position after lung cancer surgery. Repeated measures analysis of variance was used to analyze longitudinal HRQOL data. Post-hoc analyses were performed only if there was a significant within-group difference in the repeated measures one-way analysis of variance. All *P* values were two-sided, and *P* values < 0.05 were considered statistically significant. Statistical analyses were conducted using SPSS version 29.0 (IBM Inc., Chicago, IL, the USA).

## Results

A preoperative survey was conducted on patients scheduled for anatomical resection for lung cancer during the study period. Figure 1 shows the flowchart of the current study. After screening, 112 patients were found to be eligible for this research. Table 2 depicts the baseline characteristics of 93 patients who completed the preoperative survey (*n* = 40, the employed group and *n* = 53, the unemployed group). The employed group was more likely to be younger (*p* < 0.001) and have a lower CCI score (*p* = 0.017) than the unemployed group. There were no significant differences in terms of body mass index, %VC, %DLCO, surgical technique or approach, and pathological stage between the two groups. However, the unemployed group (*n* = 11 [20.7%]) had a lower proportion of patients with stage II or higher lung cancer than the employed group (*n* = 17 [42.5%]).

**Table 2:** Characteristics of the study participants at the preoperative survey

Variables	Overall (*n* = 93)		Employed (*n* = 40)		Unemployed or Retired (*n* = 53)		*P* value	Number of Values
Physical factors								
Age, mean ± SD	71.0 ± 8.4		65.3 ± 8.5		75.3 ± 5.2		< 0.001	93
Gender, Male, % (n)	57	(62%)	30	(75.0%)	27	(50.9%)	0.018	93
BMI, mean ± SD, kg/m^2^	22.6 ± 3.4		22.4 ± 3.1		22.7 ± 3.6		0.644	93
Pulmonary function								
%VC, mean ± SD	106.0 ± 13.6		104.9 ± 13.3		106.9 ± 13.8		0.480	93
%FEV1.0, mean ± SD	96.5 ± 17.6		91.9 ± 14.6		99.8 ± 18.9		0.031	93
%DLCO, mean ± SD	99.4 ± 25.3		97.9 ± 26.6		100.4 ± 24.5		0.636	92
Charlson Comorbidity Index								
0	46	(49.5%)	25	(62.5%)	21	(39.6%)	0.017^※^	93
1 or 2	36	(38.7%)	13	(32.5%)	22	(41.5%)		
> 2	11	(11.8%)	1	(2.5%)	10	(18.9%)		
Disease related factors								
Pathological stage								
0 or lA	39	(41.9%)	12	(30.0%)	27	(50.9%)	0.106	93
lB	26	(28.0%)	11	(27.5%)	15	(28.3%)		
llA or llB	14	(15.1%)	8	(20.0%)	6	(11.3%)		
lllA or lllB	14	(15.1%)	9	(22.5%)	5	(9.4%)		
Surgery								
Segmentectomy	14	(15.1%)	6	(15.0%)	8	(15.1%)	0.990	93
Lobectomy	79	(84.9%)	34	(85.0%)	45	(84.9%)		
Surgical approach								
VATS	16	(17.2%)	9	(22.5%)	7	(13.2%)	0.077	93
RATS	52	(55.9%)	17	(42.5%)	35	(66.0%)		
Thoracotomy	25	(26.9%)	14	(35.0%)	11	(20.8%)		
Postoperative complication	22	(23.7%)	11	(27.5%)	11	(20.8%)	0.408	93
Postoperative adjuvant chemotherapy	20	(21.9%)	13	(33.3%)	7	(13.4%)	0.047^※^	91
Life related factors								
JST-IC score-Social engagement-	2.9 ± 1.4		3.0 ± 1.4		2.8 ± 1.4		0.405	90
Education								
Elementary, Junior, High School	70	(75.3%)	28	(70.0%)	42	(79.2%)	0.306	93
Junior College, University	23	(24.7%)	12	(30.0%)	11	(20.8%)		
Annual household income								
< 3,000,000	23	(24.7%)	4	(10.5%)	19	(39.6%)	< 0.001	86
< 6,000,000	40	(43.0%)	16	(42.1%)	24	(50.0%)		
More than 6,000,000	23	(24.7%)	18	(47.4%)	5	(10.4%)		
Family type								
Living with spouse	43	(46.2%)	11	(27.5%)	32	(60.4%)	0.001^※^	92
Living with family (3 or more people)	37	(39.8%)	24	(60.0%)	13	(24.5%)		
Single	12	(12.9%)	4	(10.0%)	8	(15.1%)		

*%DLCO* % diffusing capacity for carbon monoxide, *%FEV1.0* percent predicted forced expiratory volume in one second, *JST* Japan Science and Technology Agency Index of Competence, *RATS* robot-assisted thoracic surgery, *SD* standard deviation, *VATS* video-assisted thoracic surgery, *%VC* % vital capacity

※Fisher's exact test

**Fig. 2:**
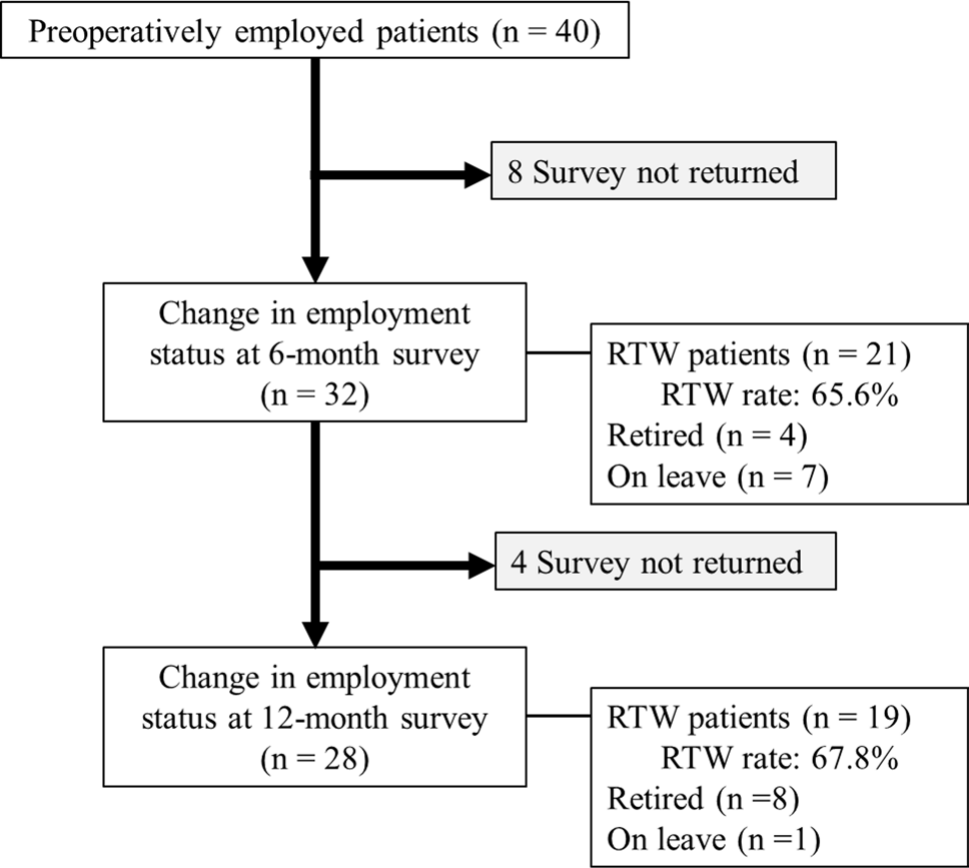
Return-to-work rates and follow-up at 6- and 12-month post-surgery

The changes in employment status after surgery are shown in Fig. 2. At 6-month post-surgery, the return-to-work (RTW) rate was 65.6%. By 12-month post-surgery, 4 patients who had been on leave at 6 months successfully returned to work, resulting in an RTW rate of 67.8%. None of the patients returned to work after retirement. Table 3 presents the employment status at 6 months postoperatively with various preoperative variables. There were no statistically significant differences in the pre- and perioperative factors between the employed and unemployed groups. Regarding employment status and living environment, the non-RTW group had a higher DL–DA in DCSQ-17 (*p* = 0.032) and a higher proportion of business owners and self-employed individuals than the RTW group (*p* = 0.048, *p* = 0.013).

**Table 3:** Comparison of pre and perioperative factors categorized by employment status at six months after surgery

Variables	RTW group		Non-RTW group		*P*-value
	(*n* = 21)		(*n* = 11)		
< Physical and treatment-related factors >					
Age, Age ± SD	62.8 ± 7.3		67.7 ± 7.8		0.096
Gender, Male, n (%)	15	(71.4%)	8	(72.7%)	0.789
BMI, mean ± SD, kg/m^2^	22.2 ± 3.2		22.8 ± 2.5		0.624
Pulmonary function					
%VC, mean ± SD	105.8 ± 12.8		105.9 ± 13.0		0.994
%FEV1.0, mean ± SD	93.1 ± 12.1		91.5 ± 17.7		0.775
%DLCO, mean ± SD	96.4 ± 26.5		94.9 ± 30.7		0.891
Charlson Comorbidity Index					
0	14	(66.7%)	7	(63.6%)	1^※^
1 or 2	6	(28.6%)	4	(36.4%)	
> 2	1	(4.8%)	0	(0%)	
< Disease related factors >					
Pathological stage					
0 or lA	8	(38.1%)	3	(27.3%)	0.955
lB	6	(28.6%)	3	(27.3%)	
llA or llB	4	(19.0%)	3	(27.3%)	
lllA or lllB	3	(14.3%)	2	(18.2%)	
Surgery					
Segmentectomy	5	(23.8%)	0	(0.0%)	0.138^※^
Lobectomy	16	(76.2%)	11	(100%)	
Surgical approach					
VATS	5	(23.8%)	2	(18.2%)	1^※^
RATS	10	(47.6%)	6	(54.5%)	
Thoracotomy	6	(28.6%)	3	(27.3%)	
Postoperative complications	4	(19.0%)	4	(36.4%)	0.397^※^
Postoperative adjuvant chemotherapy	8	(38.1%)	3	(30.0%)	1^※^
< Work related factors >					
Employment type(*n* = 31)					
Self-employed	4	(19.0%)	7	(70.0%)	0.013^※^
Contract/Temporary/Non-regular/Full-time employee	17	(81.0%)	3	(30.0%)	
Job position(*n* = 30)					
General staff	15	(75.0%)	4	(40.0%)	0.048^※^
Manager	3	(15.0%)	1	(10.0%)	
Business owners	2	(10.0%)	5	(50.0%)	
Total number of employees(*n* = 29)					
More than 50	14	(66.7%)	4	(50.0%)	0.443^※^
Industrial health consultant (*n* = 13)	2	(22.2%)	1	(25.0%)	1^※^
DCSQ-17					
PD	11.1 ± 4.1		10.0 ± 3.7		0.498
DL-SD	8.8 ± 3.0		10.4 ± 2.9		0.206
DL-DA	4.9 ± 2.4		6.8 ± 1.9		0.032^※^
SS	20.4 ± 3.0		19.3 ± 4.7		0.491
< Life related factors >					
JST-IC score-Social engagement-	3.4 ± 1.1		2.8 ± 1.3		0.224
Education					
Elementary, Junior, High School	14	(66.7%)	7	(63.6%)	1^※^
Junior College, University	7	(33.3%)	4	(36.4%)	
Annual household income					
< 3,000,000	2	(10.0%)	1	(10.0%)	0.158^※^
< 6,000,000	7	(35.0%)	7	(70.0%)	
More than 6,000,000	11	(55.0%)	2	(20.0%)	
Family type					
Living with spouse	4	(20.0%)	3	(27.3%)	0.841^※^
Living with family (3 or more people)	15	(75.0%)	7	(71.0%)	
Single	1	(5.0%)	1	(9.1%)	

*DL* Decision latitude (-*SD* Skill discretion, -*DA* Decision authority); *%DLCO* % diffusing capacity for carbon monoxide, *%FEV1.0* percent predicted forced expiratory volume in one second, *PD*: Psychological demands, *SS*: Social support, *%VC* % vital capacity

※Fisher's exact test

**Fig. 3:**
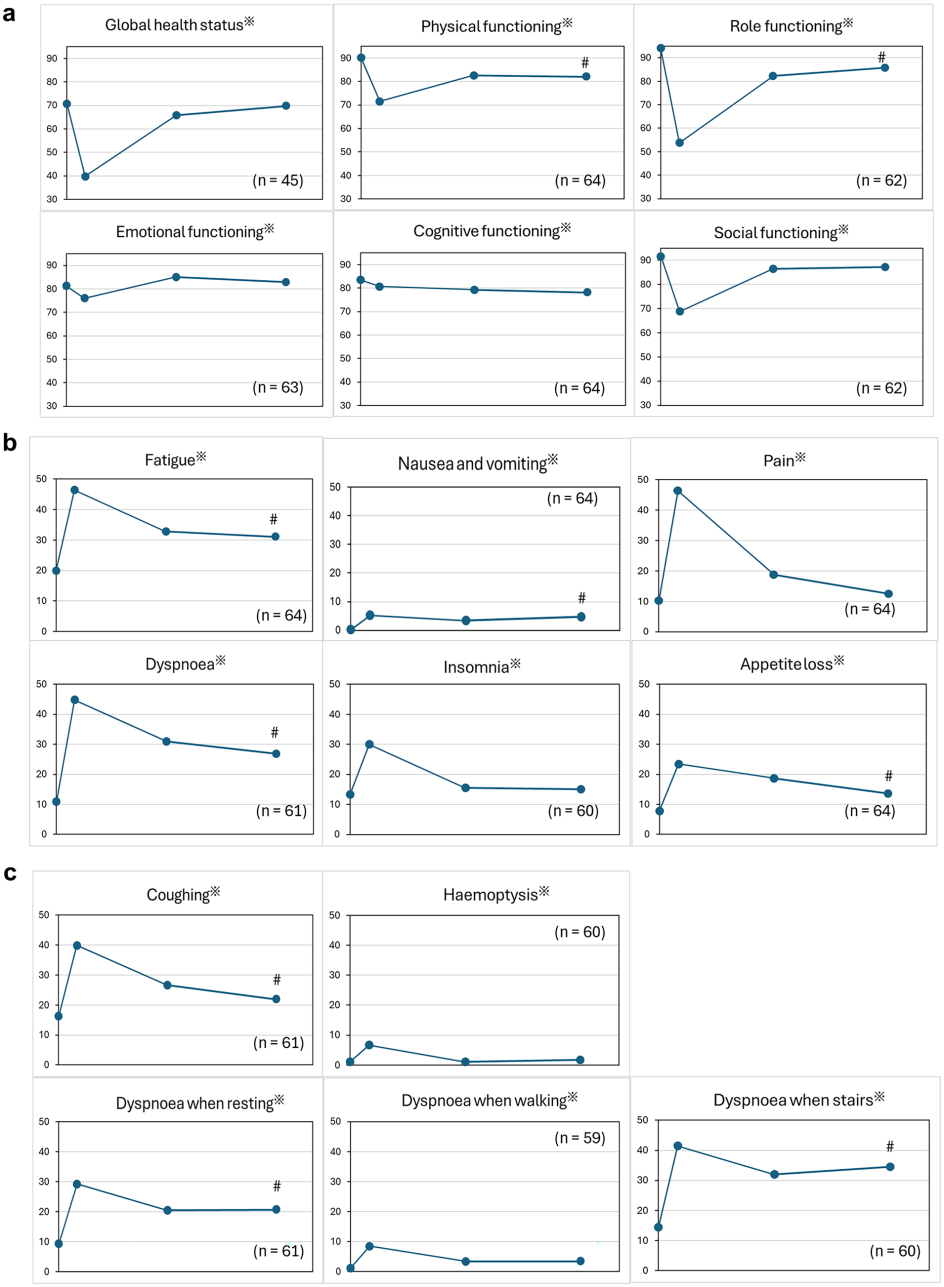
Perioperative changes in EORTC QLQ–C30 and LC-13 scores. Repeated measures analysis of variance revealed significant within-group differences, with an asterisk (*) added to the domain titles of subscores showing significance. Post hoc analyses were performed to compare baseline and 1-year postoperative values. The timing of measurement is four times: before surgery, Initial outpatient visit, 6 months after surgery, and 12 months after surgery. Symbol (#) was added to the 12 months after surgery score when it did not recover to the same level of the preoperative score. **a** Functional scale of the EORTC QLQ–C30, **b** symptom scale of the EORTC QLQ–C30, **c** Respiratory symptom scale of the EORTC QLQ–LC13

Figure 3 shows time-dependent changes in the mean QLQ–C30 and LC13 scores. Figure 3a presents the global health status and five functional scales of the QLQ–C30. Except for cognitive function, five items had a V-shaped recovery curve with the first postoperative visit as the bottom, thereby indicating within-group differences. However, the scores for physical function and role function 1 year after the surgery did not return to preoperative levels, and they differed significantly. Figure 3b depicts the symptom scale of the EORTC QLQ–C30. Figure 3c shows the symptom scale of the EORTC QLQ–LC13. All subscales of the figures exhibited significant within-group differences. The scores for fatigue, nausea and vomiting, dyspnea, appetite loss, coughing, and dyspnea when resting and climbing stairs 1 year after the surgery did not recover to the preoperative level.

## Discussion

This study examined changes in HRQOL and the RTW rate, as well as the ability to maintain employment and social roles, among Japanese patients who underwent lung cancer surgery.

Several studies showed that HRQOL typically reached its lowest point immediately after lung cancer surgery and gradually improved [25, 26], although the patient’s HRQOL was still lower even at 6 months postoperatively compared with that at baseline [27]. In the present study, we advanced their findings, newly showing that persistent symptoms such as fatigue, dyspnea, and cough did not return to preoperative levels even after 1 year after surgery. Notably, these changes were particularly prominent in domains related to physical health, such as respiratory symptoms, gastrointestinal issues, and overall physical functioning. This observation underscores the importance of addressing these physical health concerns comprehensively during follow-up care.

There were considerable challenges in terms of employment after lung cancer surgery. Nearly 35% of patients exited the workforce within the first 6 months, with further departures by 12 months, and none of the patients who left their jobs returned to work. There was no significant difference between the employed and unemployed groups in terms of cancer stage or type of surgery performed. However, the resignation group was less likely to receive adjuvant chemotherapy than the RTW group. This difference might be caused by the fact that the decision to proceed with adjuvant chemotherapy is made after consulting patients regarding their preferences and postoperative physical recovery. This also indicates that treatment strategies are implemented using a holistic approach, with consideration of the patient’s overall background rather than solely following a recommended protocol based on cancer stage.

Cancer survivors are at higher risk of unemployment than healthy people. The risk of retirement related to cancer therapy varies per country and type of cancer [28]. In a systematic review evaluating 13 studies, the RTW rates among cancer survivors in Japan ranged from 53.8% to 95.2%. However, only four of these studies included patients with lung cancer, representing a small sample size [12]. According to a large-scale study on RTW rates according to cancer type in major Japanese companies, patients with lung cancer were at higher risk of non-return to work compared with those with other cancer types, with a RTW rate of 75% at 1 year after taking sickness absence. However, in the study targeting employees of large companies, the average age at lung cancer diagnosis was 54.1 years [11]. Thus, RTW rates can vary significantly based on the country, cancer type, and characteristics of the patients.

Univariate analysis identified potential risk factors for non-RTW, including self-employment, business ownership, and a high DL–DA score. Similarly, prior studies have examined the risk factors for no-RTW across various diseases. [29, 30]. While some studies have reported self-employment as a risk factor for job loss [31, 32], others have suggested it may act as a protective fact or [31]. The influence of non-RTW risk factors likely varies depending on patient characteristics, treatment details, and countries. Therefore, hospital-based prospective data from the target population is essential to further explore these associations. A high DL–DA score, an evaluation item in the DCSQ-17, reflects a degree of decision-making authority in work tasks. The DCSQ-17 is widely used to assess occupational stress across various professions. However, no prior studies have employed this tool to investigate turnover-related factors in Japan. In general, self-employed individuals exhibit greater decision-making authority in their work [32]. In the present study, this characteristic of self-employment may have been identified as a contributing factor.

This study has several limitations. First, most patients received postoperative follow-up at other local hospitals, and few continued outpatient visits at our institution. Therefore, mail surveys were used for the 6- and 12-month follow-ups Thus, in the present study, we focused on maintaining a certain amount of response rates, but consequently, we could not assess postoperative pulmonary function. Second, the low number of employed patients at baseline limited the study's capacity to perform multivariate analysis of job loss risk factors due to insufficient statistical power, which made it difficult to draw robust scientific findings and conclusions. Third, the mean age of the study population was 71 years, exceeding the typical working age. This raised uncertainty as to whether job loss was attributed to treatment-related symptoms or if some patients had planned retirement prior to treatment. Fourth, a considerable number of patients were lost-to-follow-up in this study, which may have led to an overestimation of the reported RTW rate. Fifth, information about a perioperative support center or other organization intervening before surgery was not available in the present study. The present findings would be useful in designing the content of perioperative support. Finally, due to the small sample size, postoperative HRQOL differences arising from cancer stages or surgical techniques could not be examined.

In conclusion, this study highlights the multifaceted challenges faced by patients undergoing lung cancer surgery, particularly regarding the incomplete recovery of HRQOL and the substantial barriers to RTW. Despite advancements in perioperative care and treatments improving survival rates, persistent symptoms significantly impact patients’ physical functioning and employment outcomes. These findings emphasize the necessity for comprehensive follow-up care and tailored interventions to address both physical recovery and employment support.

## Data Availability

The datasets generated during the current study are available from the corresponding author on reasonable request. We would like to add the above sentence.
